# Validity of the Fagerström test for nicotine dependence among persons with intellectual disabilities: Cross-sectional evidence from the POWER PID study

**DOI:** 10.18332/tpc/221010

**Published:** 2026-07-26

**Authors:** Christian Ben Lakhdar, Sophie Massin, Morgane Le Blanc, Agnès d'Arripe, Cédric Routier

**Affiliations:** 1 School of ManagementUniversity Lille, CNRSVilleneuve d’AscqFrance; 2 I.D.E. Dispositif d’habitat La LiovetteBeauvaisFrance; 3 HADéPas, Institut Catholique de LilleLilleFrance

**Keywords:** intellectual disabilities, tobacco dependence, Fagerström test, exhaled carbon monoxide, vulnerable populations

## Abstract

**Introduction:**

Tobacco use and the screening of nicotine dependence using the Fagerström test among adults with intellectual disabilities (ID) is an understudied public health issue. Despite lower reported prevalence relative to the general population, methodological challenges – particularly the suitability of self-reported tools – limit reliable measurement. This study assesses the convergent validity of the Fagerström test for nicotine dependence (FTND) by comparing it with exhaled carbon monoxide (CO), an objective biomarker of recent smoking, in a sample of adults with ID.

**Methods:**

Data were collected from an observational cross-sectional validation study during 2024, which investigates addictive behaviors, health indicators, and physical activity among adults with ID in northern France. Nurses administered a standardized questionnaire, including the FTND, and measured exhaled CO using a portable analyzer. Associations were examined using correlation analyses and univariable and multivariable linear regressions controlling for sociodemographic and health variables.

**Results:**

Among 35 people who smoke, FTND scores were positively and strongly correlated with exhaled CO (r=0.540, p<0.001). In adjusted regression models, FTND remained a significantly associated factor of CO levels (0.60; 95% CI: 0.17–1.02), alongside age (0.065; 95% CI: 0.00–0.13), employment in sheltered workshops (1.86; 95% CI: -0.07–3.78), and cognitive support needs (-1.62; 95% CI: -3.51–0.26). Analyses conducted on the full sample, including 68 people who do not smoke, yielded consistent results, with FTND remaining the strongest predictor of exhaled CO.

**Conclusions:**

The results suggest evidence of validity of the FTND in adults with ID, suggesting that self-report instruments can reliably estimate nicotine dependence when administered with appropriate support. Nevertheless, the interpretation of CO levels in adults with ID should account for age, cognitive support needs, and contextual factors.

## Introduction

Tobacco use continues to be one of the leading causes of preventable morbidity and mortality worldwide, accounting for more than eight million deaths annually^1^. People with intellectual disabilities are disproportionately exposed to health inequalities, including reduced access to prevention and increased vulnerability to risk factors such as tobacco use. Although adults with intellectual disabilities (ID) have often been portrayed as having lower substance use prevalence than the general population^2,3^, emerging research challenges this assumption and highlights significant gaps in epidemiological knowledge^4^.

In France, recent studies have shed new light on tobacco use among adults with ID. The ‘Health Thermometer’, a regional survey based on Easy-to-Read and Easy-to-Understand tools conducted in the Hauts-de-France region, showed that 20.4% of adults with ID, smoke daily^5,6^. Notably, quantities consumed were comparable to those reported by the general population, underscoring that tobacco use is not only present but possibly underestimated within this group.

These findings raise important public health concerns because adults with ID frequently face barriers to prevention messages, reduced access to cessation support, and greater vulnerability to health inequalities^7^. Accurately assessing nicotine dependence in this population is therefore essential to inform tailored interventions and improve France’s efforts toward reducing tobacco-related disparities.

The Fagerström test for nicotine dependence (FTND) is one of the most widely used instruments for assessing dependence severity. While it has been validated across multiple populations and settings^8-11^, its applicability in adults with ID remains insufficiently explored. Level of support needs related to cognitive functioning, comprehension difficulties, and challenges with temporal concepts may affect response accuracy, potentially reducing the reliability of self-report measures. In contrast, exhaled carbon monoxide (CO) offers an objective biomarker of recent smoking exposure and is widely used in tobacco cessation and dependence assessment^8^. In the general population, FTND and CO typically show moderate to strong correlations^12,13^, supporting the FTND’s validity. In this context, validating the FTND against an objective biomarker such as exhaled CO is particularly important, as it allows assessing whether self-reported dependence measures remain reliable despite potential communication and comprehension challenges. To our knowledge, no study to date has empirically evaluated whether the FTND reliably measures nicotine dependence among adults with ID, highlighting the need for validation in this population.

In the context of France’s national tobacco control agenda – which emphasizes the reduction of health inequalities and improved access to adapted prevention tools for people with disabilities – validating dependence assessment instruments is a crucial step. Accordingly, this study aims to evaluate the convergent validity of the FTND by comparing its scores with exhaled CO.

## Methods

### Study design and setting

The study uses a cross-sectional observational design and draws on data collected within the emPOWERment PID (Persons with Intellectual Disabilities) project, a multisite participative research program conducted in the Hauts-de-France region (northern France). Participants were recruited using a convenience sampling strategy within partner institutions involved in the POWER PID project. Data were collected between June and September 2024 across two health and social care institutions located in Dunkerque and Beauvais. Participating structures comprised residential facilities, supported living services, and sheltered work environments providing accommodation and community-based support to adults with ID. These services included residential settings offering individualized apartments with varying levels of daily support, as well as community-based services supporting independent living and social participation. Many participants were also engaged in sheltered work environments or had previous experience in such settings, reflecting diverse levels of autonomy and support needs within the study population. Overall, these two institutions provide care and support to more than 3000 adults with ID, primarily persons with mild to moderate levels of cognitive support needs. Data were collected individually in quiet, familiar rooms within each participating structure to ensure comfort, privacy, and proper understanding.

### Participants

Participants were adults with ID living in the participating care and support services described above. All participants were aged ≥18 years and were receiving support within these services. Inclusion required the ability to participate in an oral questionnaire with communication support, including the use of visual aids such as photographs and drawings, to be enrolled in one of the partner care structures involved in the project, and to provide informed consent to participate.

### Ethics

The study adhered to French ethical and regulatory requirements for research involving human participants. Specifically, the project complies with the provisions of the Jardé Law (n° 2012–300), which regulates interventional and non-interventional research involving human subjects. The present study falls under the category of non-interventional research, implying minimal risk.

Given the cognitive support needs profiles of participants, a specific consent procedure was implemented. After a presentation of the research in simple and appropriate language, participants received an Easy-to-Read and Easy-to-Understand information sheet describing the study’s purpose, procedures, and rights. An easy-to-read consent form was read aloud and explained by trained professionals. Participants were encouraged to ask questions; clarification was provided as needed. Each participant signed the easy-to-read consent form when they felt comfortable doing so. For individuals under legal protection, the legal guardian also received an easy-to-read adapted information sheet and signed a parallel consent form, in addition to the participant’s own consent. This dual-signature procedure aims to support informed decision-making and respect participants’ autonomy, in accordance with French legislation. Participant confidentiality was strictly ensured. All data were anonymized at the time of collection and processed in compliance with General Data Protection Regulations^14^.

### Data collection procedure

All data were collected face-to-face by the nurses employed within the participants’ usual care services. This choice was intentional for several reasons. Nurses knew the participants and were trained in communication strategies adapted to ID. Their presence reduced stress, facilitated understanding, and ensured consistent questionnaire administration. The nurses received standardized instructions and a short training to harmonize questionnaire delivery and CO measurement procedures.

### Measures

Nicotine dependence was assessed using the six-item FTND^9^. Because many adults with ID may encounter difficulties interpreting written questionnaires, the instrument was administered orally, with adaptations based on Easy-to-Read and Easy-to-Understand principles: reformulated questions using simplified syntax, additional explanations when needed, pictograms illustrating key concepts, and verification of understanding before recording responses. Possible FTND scores range from 0 to 10.

Exhaled CO was measured using a portable monitor. Participants followed standardized procedures: a 15 s breath-hold followed by slow and complete exhalation. CO levels were expressed in parts per million (ppm), providing an objective indication of recent smoking exposure.

Other sociodemographic and health variables were also collected. Sociodemographic and contextual variables included: age, gender (male, female, neither), type of living arrangement (alone, with family, with a partner, in supported housing), and work status (employment or not in sheltered work settings). Living arrangements and sheltered work environments refer to structured support settings where daily activities, social interactions, and health-related behaviors, including smoking opportunities, may be influenced by organizational routines and supervision.

Concerning health variables, body mass index (BMI, kg/m²) was calculated using participants’ weight and height^15^. We also integrated items from the Washington Group Short Set (WG-SS) and its extended modules on functioning. The Washington Group tools are widely used internationally to measure disability in a standardized and inclusive way, with an emphasis on accessibility and clarity of questions. It uses a standardized four-level ordinal response scale (‘no difficulty’, ‘some difficulty’, ‘a lot of difficulty’, ‘cannot do at all’) to assess participation-relevant functional limitations. In the present study, the WG-SS served as a conceptual and linguistic foundation for constructing the adapted questionnaire, ensuring an alignment with international disability research standards, the use of simple, universal formulations, consistency with functional domains relevant to adults with ID, and compatibility with Easy-to-Read and Easy-to-Understand principles. We constructed a set of dichotomous variables in line with the WG recommendations, with ‘a lot of difficulty’ or ‘cannot do at all’ used as the threshold for functional limitation.

These variables were included to control for potential confounders known to influence exhaled CO and the pulmonary capacities^15,16^.

### Statistical analysis

Statistical analysis was performed using Stata software. A complete-case approach was used, excluding participants with missing values (listwise deletion). Descriptive statistics characterized the sample. A Spearman rank correlation test was used to quantify the association between FTND score and CO level. A univariable linear regression estimated the unadjusted FTND-CO association. Multivariable linear regression models included FTND and additional covariates to evaluate the robustness of the association and identify additional factors influencing CO levels. Analyses primarily focused on the subsample of people who smoke; however, for descriptive and population-level purposes, they were also replicated in the full sample, including people who do not smoke, in order to capture both smoking status and dependence intensity effects. Given the small size of our samples, particularly the subsample of people who smoke, and the associated risk of overfitting, we report several model specifications and examine the stability of the FTND coefficient across models. To account for potential heteroskedasticity and violations of the normality assumption, we estimated all models using heteroskedasticity-robust standard errors. The normality and independence of residuals were checked graphically, with no deviations detected. Significance was set at α=0.05.

Covariates were selected based on prior literature and their potential to confound the relationship between the FTND score and CO levels. The first category of covariates is physical in nature – gender, age, BMI, and disability-related physical condition – all of which may be associated with smoking behavior and affect exhaled CO levels through physiological mechanisms. The second category includes living situation, work status, and cognitive limitations, which may influence participants’ responses to the questionnaire and their performance on the exhaled CO test.

### Data quality and reporting standards

Data quality was ensured through standardized data collection procedures. All questionnaires were administered face-to-face by trained nurses using harmonized instructions, and consistency checks were performed during data entry to identify missing or inconsistent responses. The study is reported in accordance with the STROBE (Strengthening the Reporting of Observational Studies in Epidemiology) guidelines for cross-sectional studies.

## Results

A total of 112 individuals participated in the data collection. After exclusion of incomplete cases (3 missing exhaled CO measurements and 6 missing FTND scores among people who smoke), 103 participants, including 35 people who smoke, were included in the final analyses.

### Sample characteristics

Table 1 reports descriptive statistics for the full sample (n=103) and for the subsample of people who smoke (n=35). The full sample includes a majority of men (59.2%) and has a mean age of 41.2 years. Most participants live accompanied (62.1%) and are employed in sheltered workshops (85.4%).

**Table 1:** Characteristics of participants, Hauts-de-France region, 2024 (N=103)

Variables	Smokers		All	
	%	n	%	n
**Gender**		35		103
Male	62.9		59.2	
Female	37.1		40.8	
**Age** (years), mean (SD) [range]	42.7 (12.8) [21–64]	34	41.2 (12.8) [20–66]	99
**Living situation**		35		103
Alone	51.4		37.9	
Accompanied	48.6		62.1	
**Works in sheltered workshop**		35		103
No	14.3		14.6	
Yes	85.7		85.4	
**Smokes daily**		35		
No	20.0			
Yes	80.0			
**FTND score**, mean (SD) [range]	3.1 (2.7) [0–9]	35	1.0 (2.2) 2.0 [0–9]	103
**Exhaled CO level** (ppm), mean (SD) [range]	3.3 (2.6) [0.3–8.8]	35	1.6 (2.0) [0.2–8.8]	103
**BMI** (kg/m^2^), mean (SD) [range]	26.7 (6.8) [18.3–43.2]	35	30.6 (8.3) [17.0–63.4]	103
**Mobility limitation**		35		103
No	85.7		81.6	
Yes	14.3		18.4	
**Cognitive limitation**		35		98
No	77.1		78.6	
Yes	22.9		21.4	
**Upper body limitation**		35		102
No	91.4		87.3	
Yes	8.6		12.7	
**Anxiety or depression**		34		96
No	58.8		68.8	
Yes	41.2		31.2	
**Pain**		34		102
No	73.5		78.4	
Yes	26.5		21.6	
**Fatigue**		35		103
No	62.9		61.2	
Yes	37.1		38.8	

BMI: body mass index; CO: carbon monoxide; FTND: Fagerström test for nicotine dependence.

The subsample of people who smoke is composed of 62.9% men, with a mean age of 42.7 years. A higher proportion of people who smoke live alone compared with the full sample (51.4% vs 37.9%), while employment in sheltered workshops is similarly prevalent (85.7%).

Among people who smoke, 80% report daily tobacco use. The mean FTND score in this group is 3.1 (SD=2.7), with observed values ranging from 0 to 9. Exhaled CO levels range from 0.3 to 8.8 ppm among people who smoke (mean=3.3 ppm, SD=2.6), compared with 0.2 to 8.8 ppm in the full sample (mean=1.6 ppm, SD=2.0).

Mean BMI (kg/m^2^) is lower in the subsample of people who smoke (26.7, SD=6.8) than in the full sample (30.6, SD=8.3). The prevalence of cognitive support needs, mobility, and upper body limitations is similar across groups. Anxiety or depression is reported by 41.2% of people who smoke and 31.2% of the full sample, while pain and fatigue show comparable distributions.

### Correlation between FTND and exhaled CO

**Figure 1:**
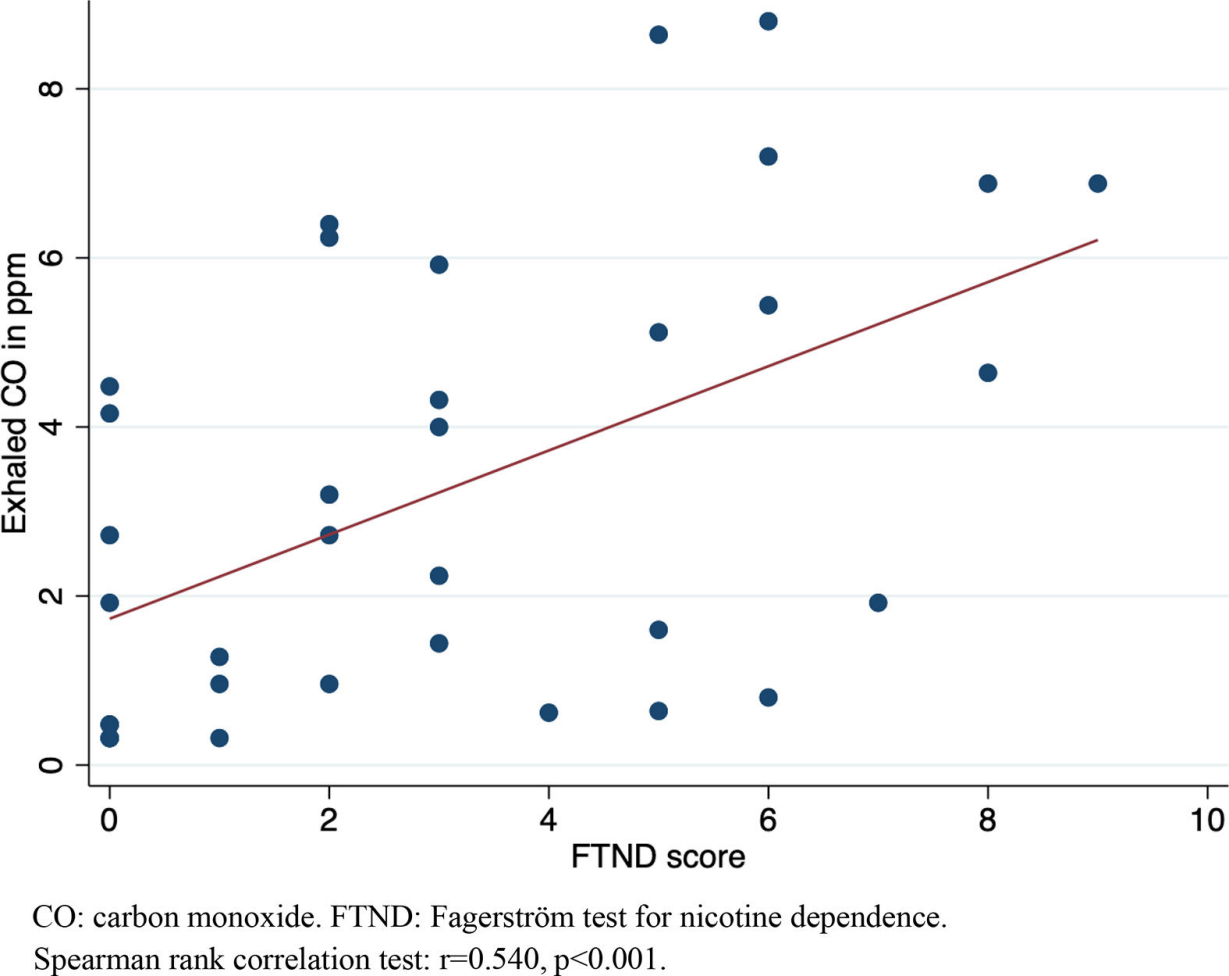
Correlation between FTND score and exhaled CO among people who smoke, Hauts-de-France region, 2024 (N=35)

A strong positive correlation was observed between FTND scores and exhaled CO (r=0.540, p<0.001). Participants with higher FTND scores systematically exhibited higher CO levels (Figure 1).

### Regression analysis

Tables 2 and 3 present the results of linear regression analyses assessing the association between FTND scores and exhaled CO levels. The FTND score significantly predicted exhaled CO in all models (Tables 2 and 3), explaining nearly one-quarter of the variance in the sample of people who smoke and half of the variance in the full sample. Age and working in a sheltered workshop were the only significant covariates in models including sociodemographic controls (columns 2). Older participants (0.065; 95% CI: 0.003–0.127) and those working in a sheltered workshop (1.857; 95% CI: -0.069–3.783) tended to have higher CO levels. Cognitive support needs were the only significant covariate in models including health control variables: participants with cognitive support needs tended to have lower CO levels (-1.624; 95% CI: -3.511–0.262). The inclusion of control variables improved the model fit, but did not alter the primary FTND-CO association. These results remained consistent when analyses were extended to the full sample, including non-smokers (Table 3).

**Table 2:** Regression results for smokers , Hauts-de-France region, 2024

Explained variable: exhaled CO in ppm	Model 1^d^	Model 2^d^	Model 3^d^
**FTND score**	0.498^c^	0.393^c^	0.596^c^
	(0.130)	(0.120)	(0.202)
	[0.234–0.762]	[0.147–0.639]	[0.174–1.019]
**Female** (ref. male)		−0.957	−0.068
		(0.847)	(1.116)
		[-2.692–0.777]	[-2.405–2.269]
**Age** (years)		0.065^b^	0.058
		(0.030)	(0.041)
		[0.003–0.127]	[-0.027–0.143]
**Lives accompanied** (ref. lives alone)		−0.375	0.082
		(0.797)	(0.834)
		[-2.008–1.258]	[-1.664–1.827]
**Works in sheltered workshop** (ref. does not work)		1.857^a^	1.758
		(0.940)	(1.330)
		[-0.069–3.783]	[-1.027–4.542]
**BMI** (kg/m^2^)			−0.010
			(0.078)
			[-0.174–0.153]
**Mobility limitation** (ref. no limitation)			−1.756
			(1.670)
			[-5.252–1.739]
**Cognitive limitation** (ref. no limitation)			−1.624^a^
			(0.901)
			[-3.511–0.262]
**Upper body limitation** (ref. no limitation)			1.544
			(1.408)
			[-1.403–4.491]
**Anxiety or depression** (ref. no anxiety or depression)			−0.618
			(0.895)
			[-2.491–1.254]
**Pain** (ref. no pain)			−0.424
			(1.177)
			[-2.888–2.040]
**Fatigue** (ref. no fatigue)			−1.451
			(1.127)
			[-3.810–0.908]
**Constant**	1.731^c^	−1.691	−0.739
	(0.457)	(1.949)	(4.480)
	[0.801–2.662]	[-5.683–2.301]	[-10.115–8.638]
**Observations**	35	34	32
R^2^	0.264	0.395	0.618

Linear regression: beta coefficients, robust standard errors in parentheses, and 95% confidence intervals in brackets.

a p<0.1

b p<0.05

c p<0.01.

d Model 1: unadjusted linear regression including FTND score only. Model 2: adjusted for sociodemographic variables (gender, age, living arrangement, and employment in sheltered workshop). Model 3: additionally adjusted for health-related variables (BMI, mobility limitation, cognitive limitation, upper body limitation, anxiety or depression, pain, and fatigue). BMI: body mass index. CO: carbonmonoxide. FTND: Fagerström test for nicotine dependence. ppm: parts per million.

**Table 3:** Regression results for the full sample, Hauts-de-France region, 2024

Explained variable: exhaled CO in ppm	Model 1^d^	Model 2^d^	Model 3^d^
**FTND score**	0.648^c^	0.631^c^	0.621^c^
	(0.104)	(0.099)	(0.102)
	[0.443–0.854]	[0.433–0.828]	[0.418–0.825]
**Female** (ref. male)		−0.206	−0.194
		(0.274)	(0.323)
		[-0.750–0.339]	[-0.837–0.450]
**Age** (years)		0.025^a^	0.023^a^
		(0.014)	(0.013)
		[-0.002–0.052]	[-0.003–0.049]
**Lives accompanied** (ref. lives alone)		0.197	0.126
		(0.307)	(0.315)
		[-0.413–0.806]	[-0.503–0.754]
**Works in sheltered workshop** (ref. does not work)		0.594	0.683^a^
		(0.464)	(0.389)
		[-0.328–1.515]	[-0.092–1.459]
**BMI** (kg/m^2^)			−0.031
			(0.019)
			[-0.069–0.007]
**Mobility limitation** (ref. no limitation)			−0.237
			(0.554)
			[-1.341–0.867]
**Cognitive limitation** (ref. no limitation)			−0.812^b^
			(0.374)
			[-1.557–−0.068]
**Upper body limitation** (ref. no limitation)			0.652
			(0.537)
			[-0.417–1.721]
**Anxiety or depression** (ref. no anxiety or depression)			0.213
			(0.368)
			[-0.521–0.946]
**Pain** (ref. no pain)			−0.288
			(0.489)
			[-1.262–0.687]
**Fatigue** (ref. no fatigue)			−0.630
			(0.405)
			[-1.438–0.177]
**Constant**	0.915^c^	−0.635	0.810
	(0.101)	(0.889)	(1.001)
	[0.716–1.115]	[-2.400–1.130]	[-1.185–2.805]
**Observations**	103	99	88
**R^2^**	0.496	0.518	0.598

Linear regression: beta coefficients, robust standard errors in parentheses, and 95% confidence intervals in brackets.

a p<0.1

b p<0.05

c p<0.01.

d Model 1: unadjusted linear regression including FTND score only. Model 2: adjusted for sociodemographic variables (gender, age, living arrangement, and employment in sheltered workshop). Model 3: additionally adjusted for health-related variables (BMI, mobility limitation, cognitive limitation, upper body limitation, anxiety or depression, pain, and fatigue). BMI: body mass index. CO: carbonmonoxide. FTND: Fagerström test for nicotine dependence. ppm: parts per million.

## Discussion

This study provides evidence that the Fagerström test for nicotine dependence is a valid tool for assessing nicotine dependence among adults with ID. The strong positive correlation and robust regression results confirm the test’s convergent validity relative to exhaled CO.

### Interpretation of findings

The association between FTND and CO in this sample is comparable to correlations observed in general population studies in Tunisia^12^ or in India^13^. These comparisons should be interpreted with caution, as previous studies relied on larger samples of people who smoke from the general population and were conducted in different socio-cultural contexts, which may limit direct comparability. This is, however, notable given the potential challenges in using self-reported tools among individuals with cognitive support needs. The successful administration of the FTND using easy-to-read adapted procedures likely contributed to this reliability.

Previous studies in the general population have shown that exhaled carbon monoxide levels are influenced by a range of physiological and behavioral factors beyond smoking intensity. In particular, lung diffusion capacity and CO clearance have been shown to vary with body composition and obesity-related^17^ respiratory mechanics, physical fitness, mobility, and exercise levels, as well as with sex-related differences in CO elimination kinetics^18-22^. These factors may affect the accumulation and elimination of CO independently of nicotine dependence and are therefore important potential confounders when interpreting CO measurements.

In contrast, our findings suggest a somewhat different pattern in adults with ID. After controlling for a broad set of health and functional variables, age, employment in sheltered workshops, and cognitive support needs were significantly associated with exhaled CO levels. Older participants tended to exhibit higher CO levels, which may be associated with longer smoking histories or differences in physiological clearance. Similarly, employment in sheltered workshops was associated with higher CO levels, which could be related to contextual or organizational factors such as smoking opportunities, daily routines, or social norms within these environments.

Finally, participants with cognitive support needs showed lower CO levels, which may reflect differences in smoking patterns, consumption intensity, supervision, or reporting accuracy. These results suggest that, in adults with ID, social context and cognitive support needs may play a more prominent role in shaping CO exposure than the physiological determinants typically emphasized in the general population literature.

### Implications for practice

The findings of this study suggest that the FTND may be a useful tool for assessing nicotine dependence among adults with ID, provided that it is administered using adapted communication strategies. In this study, the use of simplified language and visual supports appeared to facilitate understanding and consistent responses, which may help improve the feasibility of self-reported measures in this population.

In addition, the observed convergence between FTND scores and exhaled CO indicates that combining self-reported and objective measures could provide a more comprehensive assessment of smoking behavior. Such an approach may be particularly relevant in populations where communication difficulties could affect reporting accuracy.

However, these implications should be interpreted with caution, given the cross-sectional design and the relatively small sample size. Further studies, including longitudinal and interventional designs, are needed to confirm the robustness of these findings and to determine how such tools can be effectively integrated into routine practice.

### Limitations

Several limitations should be considered when interpreting the findings of this study. First, the cross-sectional design does not allow for causal inference regarding the relationship between FTND scores and exhaled CO levels. Because CO reflects recent smoking exposure, fluctuations due to timing of last cigarette, environmental exposure, or daily variability could not be fully controlled for. Second, although the FTND was administered using adapted communication strategies, the instrument was not originally designed for adults with ID, and certain items requiring temporal reasoning or self-assessment may still have been challenging for some participants. This may have introduced measurement noise and potential information bias related to self-reported data, even though this study suggests overall good convergent validity. In addition, the adapted version of the FTND was not formally piloted or validated prior to its use in this study, and the adapted version of the Washington Group Short Set (WG-SS) was not formally tested for comprehension, which may affect the reliability, comparability, and interpretability of the responses. Third, the relatively small sample size limits statistical power and the generalizability of the findings. Fourth, the study relied on a convenience sample drawn from institutions participating in the POWER PID project. As a result, the findings may not be fully generalizable to adults with ID living independently, with family, or in other regions of France. Institutional characteristics such as staff training, health culture, or smoking norms may also have influenced responses or CO levels. Fifth, information on residual confounders, such as time since the last cigarette, type of tobacco used, or secondhand smoke exposure, was not systematically collected and could not be incorporated into the analyses. Finally, although CO measurement provides an objective indicator of smoking, its accuracy may be influenced by physiological factors such as mobility, fitness level, or variations in lung function, which were only partially captured by available covariates. Despite these limitations, this study offers the first empirical evidence on the validity of the FTND in adults with ID and provides a valuable foundation for future research.

### Future research

Future research could build on these findings by replicating the analyses in larger and more diverse samples of adults with ID, including individuals living outside institutional settings. Given the cross-sectional design of the present study, longitudinal approaches would be particularly valuable to better understand the temporal relationship between FTND scores and smoking behavior, as well as their association with changes in objective biomarkers such as exhaled CO. In addition, further work may explore how adapted administration procedures influence the reliability of self-reported measures in this population. This could help clarify whether the observed validity of the FTND is partly driven by the use of tailored communication strategies. Overall, these findings should be considered preliminary and call for more robust study designs to confirm the validity and practical utility of nicotine dependence measures among adults with ID.

## Conclusions

The present study provides initial evidence that the FTND shows convergent validity with exhaled CO among adults with ID. These findings suggest that, when administered using adapted communication strategies, the FTND may offer a feasible approach to assessing nicotine dependence in this population. However, given the cross-sectional design and the limited sample size, these results should be interpreted with caution. Additional studies, particularly those using larger samples and longitudinal designs, are needed to confirm the validity and reliability of the FTND and to better understand its applicability across different contexts and subgroups of adults with ID.
